# Protrudin modulates seizure activity through GABA_A_ receptor regulation

**DOI:** 10.1038/s41419-019-2118-8

**Published:** 2019-11-27

**Authors:** Xi Lu, Yong Yang, Ruijiao Zhou, Yun Li, Yi Yang, Xuefeng Wang

**Affiliations:** 1 Department of Neurology, The First Affiliated Hospital of Nanchang University, 17 Yongwaizheng Street, Nanchang, 330006 Jiangxi China; 2 Department of Neurology, The First Affiliated Hospital of Chongqing Medical University, Chongqing Key Laboratory of Neurology, 1 Youyi Road, Chongqing, 400016 China

**Keywords:** Immunology, Synaptic transmission

## Abstract

Epilepsy is a serious neurological disease characterized by recurrent unprovoked seizures. The exact etiology of epilepsy is not fully understood. Protrudin is a neural membrane protein and is found to be mutated in hereditary spastic paraplegia that characterized by symptoms like seizures. Here, we reported that the expression of protrudin was downregulated in the temporal neocortex of epileptic patients and in the hippocampus and cortex of pentylenetetrazol and kainic acid-kindled epileptic mouse models. Behavioral and electroencephalogram analyses indicated that overexpression of protrudin in the mouse hippocampus increased the latency of the seizure and decreased the frequency and duration of seizure activity. Using whole-cell patch clamp, overexpression of protrudin in the mouse hippocampus resulted in a reduction in action potential frequency and an increase in gamma-aminobutyric acid (GABA)ergic inhibitory current amplitude. Moreover, western blot analysis showed that the membrane expression of the GABA A receptor β2/3 subunit was also upregulated after protrudin overexpression, and coimmunoprecipitation resulted in a protein–protein interaction between protrudin, GABA_A_Rβ2/3 and GABA receptor-associated protein in the hippocampus of epileptic mice. These findings suggest that protrudin probably inhibits the occurrence and development of epilepsy through the regulation of GABA_A_ receptor-mediated synaptic transmission, and protrudin might be a promising target for the treatment of epilepsy.

## Introduction

Epilepsy is a devastating neurological disease and is characterized by recurrent seizure activity. Epilepsy has a worldwide prevalence of ~ 1–2% with ~ 65 million people suffering in the world^1^. Almost 30% of newly diagnosed epileptic patients fail to respond to antiepileptic drugs and develop pharmacoresistant and intractable epilepsy^2^. As the most common form of intractable epilepsy, temporal lobe epilepsy (TLE) often results in poor prognoses^3^. Epileptic seizures are generally accepted to be attributed to an imbalance of excitatory and inhibitory synaptic transmission^2^. Nevertheless, the exact etiology and pathogenesis of epilepsy still have an incomplete understanding. Investigating the mechanisms underlying epilepsy will hopefully improve therapeutic strategies.

Protrudin (ZFYVE27, SPG33) is a neural membrane protein that regulates vesicular transport, neurite outgrowth, and endoplasmic reticulum formation in vitro^4–8^. Protrudin was first found to be mutated in a German family with hereditary spastic paraplegia (HSP). The mutated protrudin protein shows an aberrant intracellular pattern in its tubular structure and that it affects neuronal intracellular trafficking in the corticospinal tract^9^. HSP is an inherited neurodegenerative disorder characterized by progressive bilateral leg stiffness and spasticity at rest. Complex forms of the disorder are featured by additional symptoms such as seizures, dementia, and cerebellar dysfunction^10^. Epilepsies and HSP have been proposed to share a common pathogenesis theme because some HSP-related proteins are implicated in the development of epilepsy^11–13^. However, the function of protrudin in epilepsy remains unknown. We speculated that the protrudin protein may be associated with seizure activity.

In this study, we investigated the expression patterns of protrudin protein in epileptic mouse models and in patients with intractable TLE. In addition, we performed behavioral and electrophysiological analyses after lentivirus (LV)-mediated overexpression of protrudin to further explore the possible role of protrudin in epilepsy.

## Results

### Expression patterns of the protrudin protein in TLE patients

**Fig. 1:**
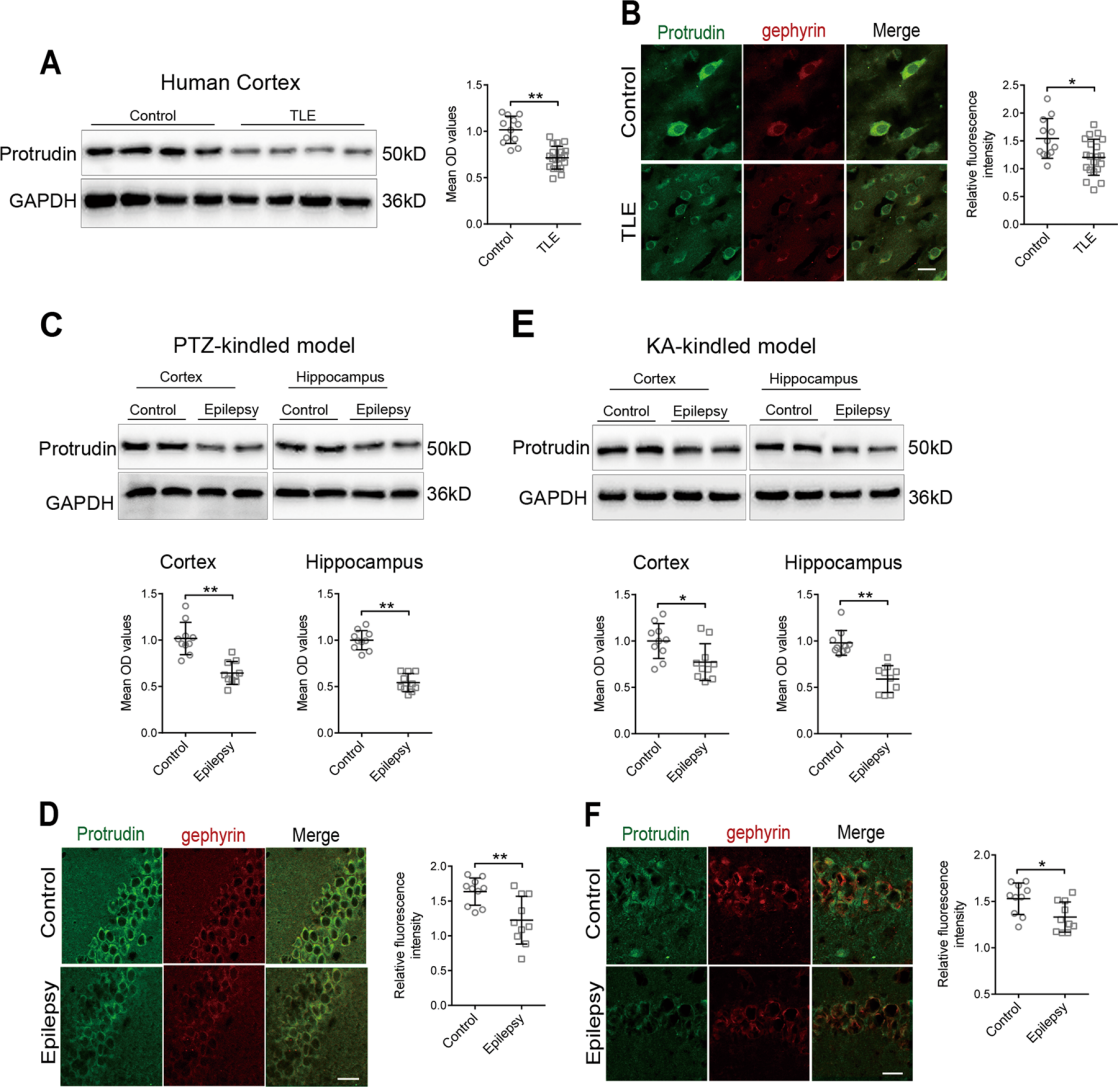
Expression of protrudin in TLE patients and epileptic mice. **a**, **b** Protrudin expression in the temporal neocortex of TLE patients and control patients by immunoblot (**a**) and immunofluorescence staining (**b**). Bar, 40 μm. **c**, **d** Protrudin expression in the hippocampus (**c**, **d**) and adjacent temporal cortex (**c**) of PTZ-induced mice. Bar, 40 μm. **e**, **f** Protrudin expression in the hippocampus (**e**, **f**) and adjacent temporal neocortex (**e**) was compared between KA-treated mice and control mice. Bar, 40 μm. Protrudin was colocalized with the inhibitory synaptic marker gephyrin (**b**, **d**, **f**). OD, optical density. Student’s *t* test, *p < 0.05, **p < 0.01.

The expression of protrudin in the temporal neocortex from TLE patients (*n* = 20) and control individuals (*n* = 12) was evaluated by western blot analysis and double immunofluorescence labeling. The expression of protrudin was significantly lower in TLE patients than in controls (Control, 1.016 ± 0.147; TLE, 0.715 ± 0.122; *P* = 6.595 × 10^−7^) (Fig. 1a). Immunofluorescence revealed that the protrudin protein was mainly found in the neuronal cytomembrane and cytoplasm, and was colocalized with the inhibitory synaptic marker gephyrin in the temporal cortex of control and TLE patients (Fig. 1b). Quantitative immunofluorescence analysis showed a low fluorescence intensity of the protrudin protein in TLE patients compared with that in the control group (Control, 1.542 ± 0.357; TLE, 1.205 ± 0.324; *P* = 0.010) (Fig. 1b).

### Expression of the protrudin protein in epileptic mouse models

To further investigate the expression of protrudin in the epileptic mouse models, we analyzed protrudin protein expression by western blot and quantitative immunofluorescence. The expression in the mouse models was similar to that found in humans. In pentylenetetrazol (PTZ)-kindled mice, protrudin protein levels were significantly reduced in both the hippocampus (Control, 1.0 ± 0.104; Epilepsy, 0.543 ± 0.098; *P* = 7.180×10^−9^) and adjacent neocortex (Control, 1.017 ± 0.174; Epilepsy, 0.645 ± 0.123; *P* = 3.131×10^−5^) compared with the levels in the corresponding controls (Control, *n* = 10; Epilepsy, *n* = 10) (Fig. 1c). Meanwhile, quantitative immunofluorescence analysis revealed only weak green staining for protrudin in the hippocampus of PTZ-kindled mice, whereas strong staining for protrudin was detected in controls (Control, 1.636 ± 0.196; Epilepsy, 1.224 ± 0.343; *P* = 0.004) (Fig. 1d).

Moreover, the expression pattern of protrudin was confirmed in kainic acid (KA)-kindled mice. The intensity ratio was significantly decreased in the hippocampus (Control, 0.978 ± 0.133; Epilepsy, 0.589 ± 0.145; *P* = 6.955×10^−6^) and adjacent temporal cortex (Control, 1.0 ± 0.189; Epilepsy, 0.773 ± 0.198; *P* = 0.017) in KA-kindled mice compared with that in controls (Control, *n* = 10; Epilepsy, *n* = 10) (Fig. 1e). Immunofluorescence staining verified a lower fluorescence intensity of protrudin protein in the hippocampus of KA-kindled mice than in that of controls (Control, 1.528 ± 0.169; Epilepsy, 1.331 ± 0.163; *P* = 0.016) (Fig. 1f).

### Identification of protrudin expression after hippocampal injection of recombinant lentivirus

**Fig. 2:**
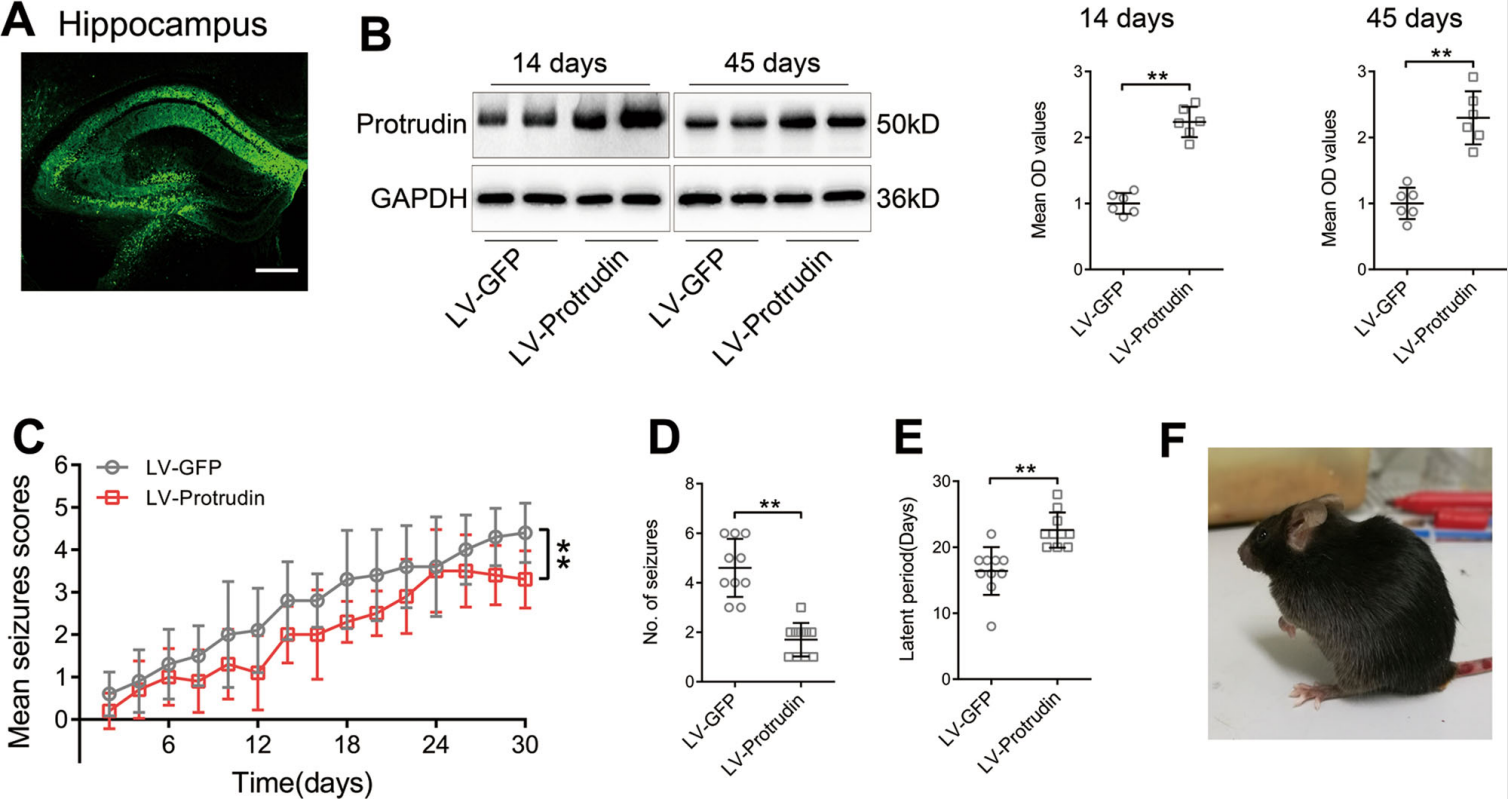
Effect of LV-protrudin on seizure susceptibility in PTZ-induced mice. **a** LV-encoded green fluorescent protein (GFP) was detected in the hippocampus following the injection of LV. Bar, 250 μm. **b** Hippocampal protrudin protein expression levels were detected in protrudin overexpressing mice 14 and 45 days after LV injection. OD, optical density. **c**–**e** Quantitative analysis of daily seizure scores (**c**), total number of seizures with a score of 4 or 5 (**d**) and duration and latency of the first seizure with a score of 4 or 5 (**e**) in PTZ-induced mice after protrudin overexpression. **f** A mouse with Racine’s stage IV seizure presented rearing and myoclonic twitching. Repeated measures ANOVA followed by post hoc *t* tests for **c**, Student’s *t* test for **b**, **d**, and **e**, **p < 0.01.

Lentivirus (LV)-protrudin was injected into the dorsal hippocampal region of the mice. The transfection efficiency was measured by detecting GFP autofluorescence staining at 14 days and 45 days after LV injection. In Fig. 2a, GFP was found in the injected hippocampus, notably in the CA3 region. Western blot showed that the expression of protrudin was significantly higher in the LV-protrudin group than in the corresponding LV-GFP control group on days 14 (LV-GFP, 1.0 ± 0.158; LV-Protrudin, 2.233 ± 0.23; *P* = 7.62×10^−7^) and 45 (LV-GFP, 1.0 ± 0.237; LV-Protrudin, 2.297 ± 0.402; *P* = 4.745×10^−5^) after LV injection (LV-GFP, *n* = 6; LV-Protrudin, *n* = 6) (Fig. 2b). These results indicated that hippocampal neurons were successfully infected by LV-protrudin, which efficiently increased protrudin expression.

### Protrudin reduces seizure susceptibility in epileptic mouse models

To further explore whether protrudin modulates epilepsy susceptibility, behavioral, and electroencephalogram analyses were performed in epileptic mouse models. Behavioral observation performed in PTZ-kindled mice showed that daily seizure scores were lower in LV-protrudin mice than in LV-GFP controls (F(1,18) = 49.779, *P* = 1.398×10^−6^) (LV-GFP, *n* = 10; LV-Protrudin, *n* = 10) (Fig. 2c). More specifically, the LV-protrudin mice exhibited a lower total number of seizures (LV-GFP, 4.6 ± 1.174; LV-Protrudin, 1.7 ± 0.675; *P* = 2.408×10^−6^) (Fig. 2d) and longer induced latency (LV-GFP, 16.4 ± 3.627; LV-Protrudin, 22.6 ± 2.67; *P* = 3.856×10^−4^) (Fig. 2e) than LV-GFP controls.

**Fig. 3:**
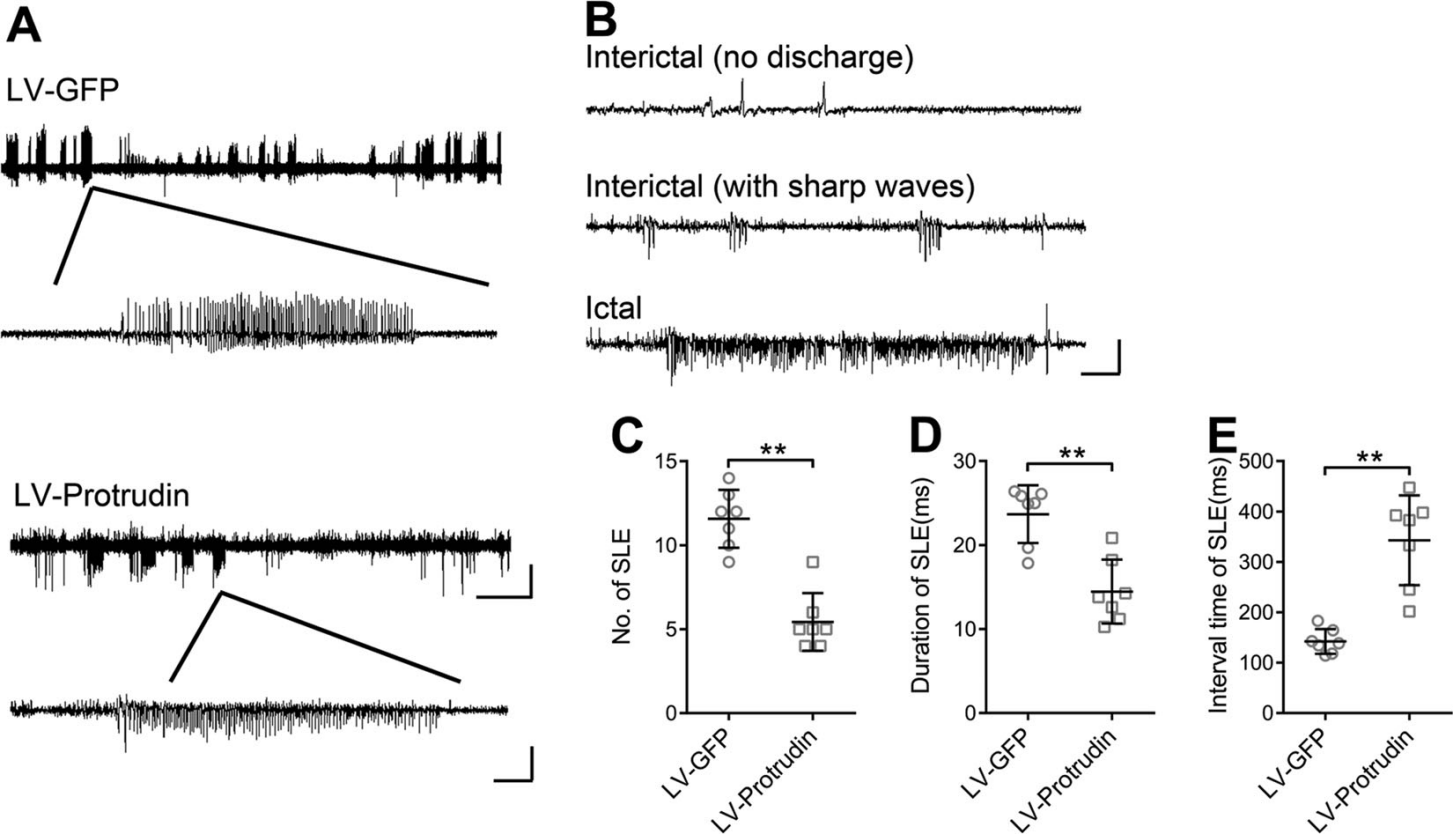
Effect of LV-protrudin on seizure susceptibility in KA-induced mice. **a** Representative images of SLEs obtained in each group during a 30 min EEG recording in the KA-induced epilepsy model. Bar (up), 1.5 mV, 200 s; Bar (down), 1.5 mV, 5 s. **b** EEG of KA-induced mice showed normal baseline, sharp waves in the interictal stage and a spontaneous recurrent seizure. Bar, 1.5 mV, 5 s. **c**–**e**. Quantitative analysis of number (**c**), duration (**d**), and interval times (**e**) of SLEs in KA-induced mice. Student’s *t* test, **p < 0.01.

In the electroencephalogram (EEG) analysis of KA-induced mice, seizure-like events (SLEs) were recorded on EEGs (Fig. 3a, b). The LV-protrudin group presented fewer SLEs (LV-GFP, 11.57 ± 1.718; LV-Protrudin, 5.429 ± 1.718; *P* = 2.233×10^−5^) and had a shorter duration of SLEs (LV-GFP, 23.68 ± 3.442; LV-Protrudin, 14.45 ± 3.809; *P* = 4.676×10^−4^) and a longer SLE interval time (LV-GFP, 142.1 ± 24.5; LV-Protrudin, 342.9 ± 89.06; *P* = 9.132×10^−5^) than the LV-GFP group (LV-GFP, *n* = 7; LV-Protrudin, *n* = 7) (Fig. 3c–e). These behavioral and electroencephalogram results suggested that protrudin reduced susceptibility to epilepsy by changing the frequency, duration, and latency of seizures.

### Effects of protrudin on inhibitory synaptic transmission

**Fig. 4:**
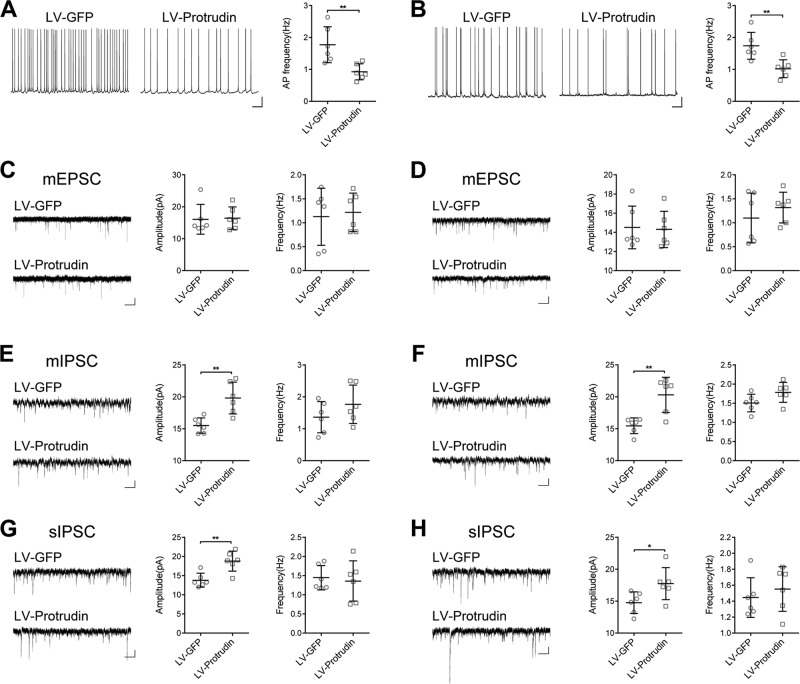
Effects of protrudin on electrophysiology in the hippocampus of PTZ- and KA-kindled mouse models. **a**, **b** The frequency of spontaneous AP was compared between the LV-protrudin group and the control group in PTZ-induced mice (**a**) and KA-kindled mice (**b**). Bar **a**, **b**, 12.5 mV, 1.25 s. **c**, **d** Protrudin had no significant effect on the amplitude or frequency of mEPSCs in PTZ- (**c**) and KA-kindled mice (**d**). **e**–**h**, Quantitative analysis of mIPSC and sIPSC amplitude and frequency in CA3 pyramidal neurons of the hippocampus between the LV-protrudin group and the control group in PTZ- (**e**, **g**) and KA-induced mice (**f**, **h**). Bar **c**–**h**, 10 pA, 1.25 s. Student’s *t* test, *p < 0.05, **p < 0.01.

Whole-cell patch clamp electrophysiology was performed in PTZ- and KA-kindled mouse models and Mg^2+^-free artificial cerebral spinal fluid (ACSF) epilepsy cell model. The Mg^2+^-free model is a classic in vitro epilepsy model that induces spontaneous epileptic form discharges^14–16^. Hippocampal CA3 area pyramidal neurons in brain slices from mice received the LV-protrudin injection were examined (*n* = 6 neurons from three mice in each group). We first analyzed the effect of protrudin on spontaneous action potentials (APs) in pyramidal neurons. The frequency of spontaneous APs in the LV-protrudin group was significantly lower than that in the LV-GFP control group in PTZ- and KA-kindled mouse models (Fig. 4a, b) and Mg^2+^-free model (Fig. S1A).

To further explore the contribution of excitatory receptors and/or inhibitory synaptic inputs to the neuronal low excitability induced by protrudin, miniature excitatory postsynaptic currents (mEPSCs), miniature inhibitory postsynaptic currents (mIPSCs), and spontaneous (s)IPSCs in pyramidal neurons of the hippocampus were recorded^17,18^. There was no significant difference in the amplitudes or frequency of mEPSCs between the LV-protrudin group and the control group in PTZ- kindled and KA-kindled mouse models (Fig. 4c, d) and Mg^2+^-free model (Fig. S1B). However, the mIPSC and sIPSC amplitudes but not the frequencies were significantly increased in LV-protrudin mice compared with those in LV-GFP controls in PTZ- and KA-kindled mouse models (Fig. 4e-h) and Mg^2+^-free model (Fig. S1C, D).

**Fig. 5:**
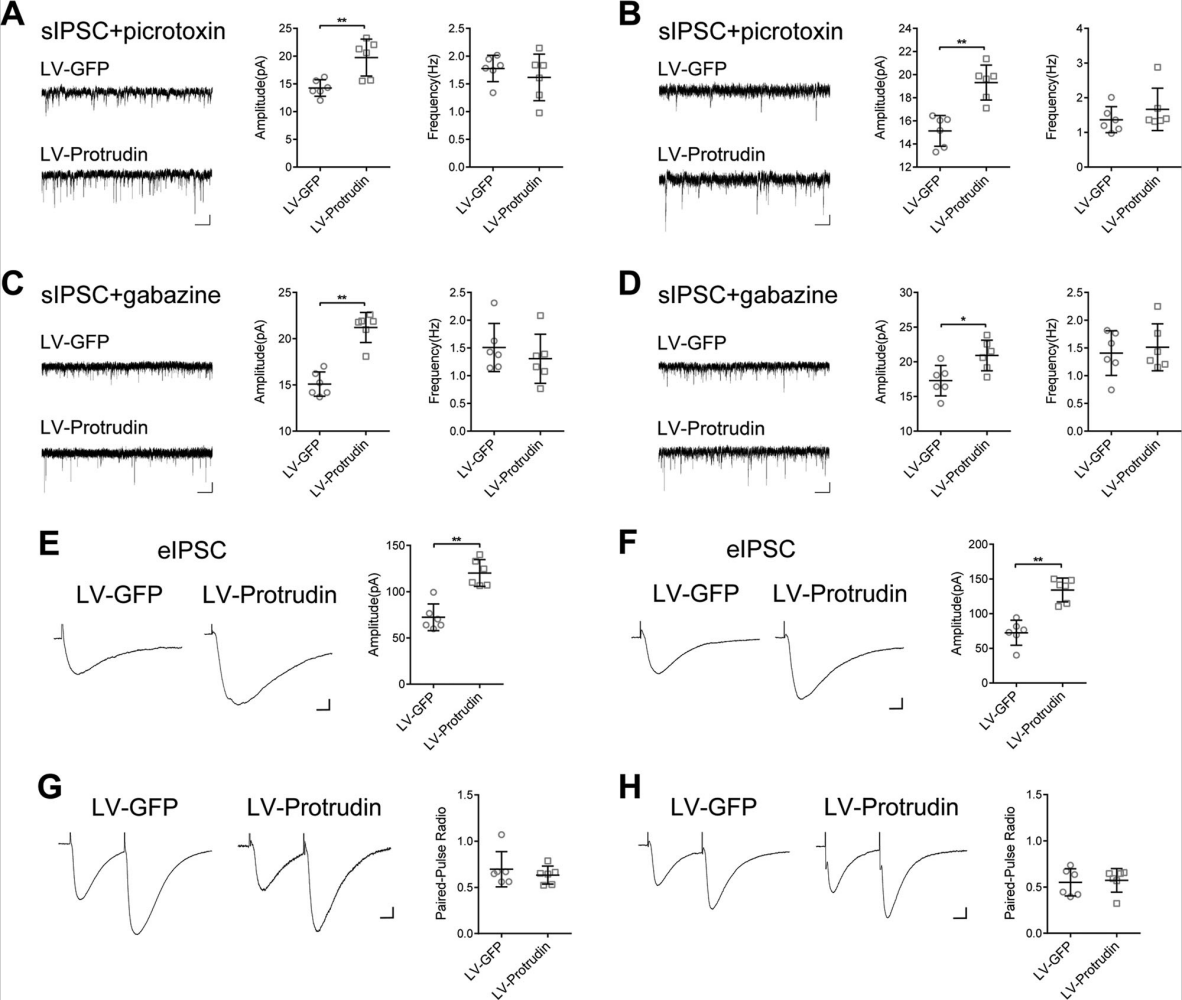
Effects of protrudin on IPSCs in the hippocampus of PTZ- and KA-kindled mice. **a**, **b** Quantitative analysis of the phasic current amplitude between the LV-protrudin group and the control group after inhibiting the tonic inhibitory currents with picrotoxin in PTZ- (**a**) and KA-induced mouse models (**b**). **c**, **d**, Quantitative analysis of tonic inhibitory current amplitude between the LV-protrudin group and the LV-GFP group after selectively blocking the phasic inhibitory currents with gabazine in PTZ- (**c**) and KA-induced mice (**d**). Bar **a**-**d**, 10pA, 1.25 s. **e**, **f**, eIPSCs from hippocampal pyramidal cells in protrudin overexpressing mice in PTZ- (**e**) and KA-induced mice (**f**). **g**, **h**, Overexpressing protrudin did not alter the paired-pulse ratio (PPR) values in PTZ- (**g**) and KA-induced mice (**h**). Bar **e**-**h**, 20pA, 12.5 s. Student’s *t* test, *p < 0.05, **p < 0.01.

Gamma-aminobutyric acid (GABA) A receptor (GABA_A_R)-mediated GABAergic inhibitory transmission is the major neural inhibition in the adult mammal brain. GABA_A_Rs generate two types of inhibitory currents: classical phasic (via synaptic receptors) and tonic inhibition (via extrasynaptic receptors)^19–21^. To clarify the effect of protrudin of synaptic origin, we recorded sIPSCs by application of a lower concentration of gabazine (0.5 μm) to selectively block the phasic inhibitory currents and picrotoxin (10 μm) to inhibit the tonic currents. In PTZ- and KA-kindled mouse models, sIPSC amplitudes were remarkably higher in the LV-protrudin group than in the controls in the presence of picrotoxin (Fig. 5a, b) and gabazine (Fig. 5c, d). Similar results were observed in Mg^2+^-free model (Fig. S1E, F). These results suggest that protrudin may both affect the phasic inhibition mediated by synaptic GABA_A_Rs and tonic inhibition via extrasynaptic receptors.

Furthermore, evoked IPSCs (eIPSCs) were detected to verify the increased mIPSC and sIPSC amplitudes. eIPSC amplitude was significantly higher in LV-protrudin mice than in control mice in PTZ- and KA-kindled models (Fig. 5e, f) and Mg^2+^-free model (Fig. S2A). Paired-pulse ratio (PPR) is a reliable index of the contribution of presynaptic vesicle release to inhibitory postsynaptic currents^22^. Consistent with the results of no change in mIPSC or sIPSC frequency, there was no significant difference in PPR between the LV-protrudin mice and LV-GFP controls (Fig. 5g, h and Fig. S2B), indicating that presynaptic vesicle release was not responsible for the protrudin-mediated changes in IPSCs in the epileptic models.

### Effect of protrudin on GABA_A_ receptors

**Fig. 6:**
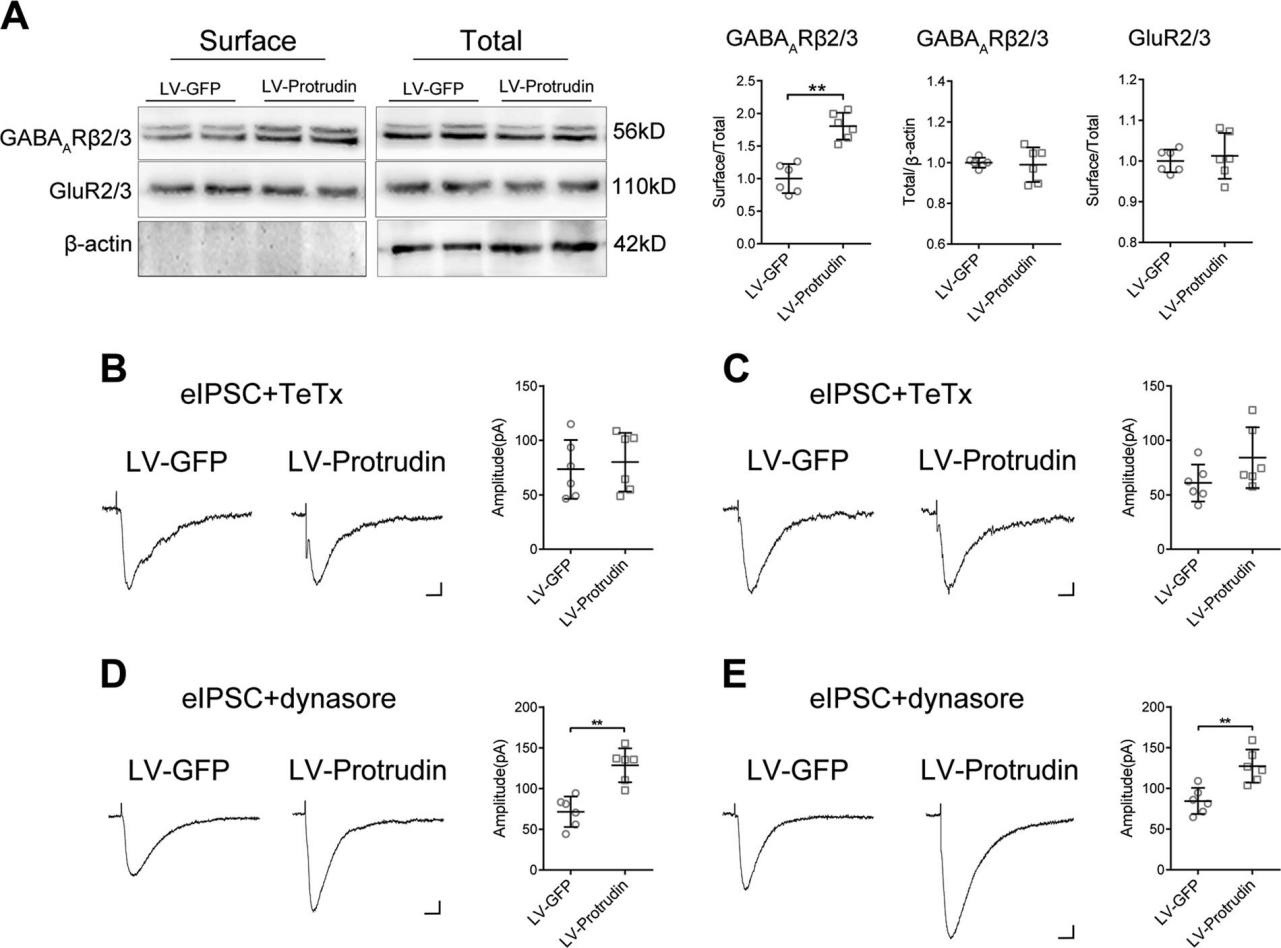
Effect of protrudin on cell surface GABA_A_ receptors. **a** Representative images of surface and total GABA_A_Rβ2/3 expression in the hippocampus of PTZ-kindled mice in the LV-protrudin group and control group. Surface GABA_A_Rβ2/3 protein expression in the LV-protrudin group was significantly higher than that in the control mice, whereas total GABA_A_Rβ2/3 protein and surface/total GluR2/3 protein expression were unchanged. **b**, **c** Representative traces of eIPSCs in the LV-protrudin group and control group after treatment with TeTx in PTZ- (**b**) and KA-kindled mice (**c**) and the summary of the amplitude. **d**, **e** Representative traces of eIPSCs in the LV-protrudin group and control group in the present of dynasore in PTZ- (**d**) and KA-kindled mice (**e**). Bar **b**–**e**, 20 pA, 12.5 s. Student’s *t* test, **p < 0.01.

Inhibitory postsynaptic transmission is mainly regulated by GABA_A_Rs^23^. To investigate the mechanism by which protrudin increases the inhibitory postsynaptic current in epilepsy, we detected the expression of GABA_A_Rβ2/3, two key subunits of GABA_A_Rs in neurons, in PTZ-induced epilepsy mice following lentiviral overexpression of protrudin. Western blot showed that there was no significant difference in the total GABA_A_Rβ2/3 protein in the epileptic hippocampus between the LV-protrudin group and the LV-GFP group (LV-GFP, 1.000 ± 0.234; LV-Protrudin, 0.99 ± 0.085; *P* = 0.788) (LV-GFP, *n* = 6; LV-Protrudin, *n* = 6) (Fig. 6a). In contrast, the cell surface expression of GABA_A_Rβ2/3 protein in the LV-protrudin group was significantly higher than that in the LV-GFP group (LV-GFP, 1.000 ± 0.223; LV-Protrudin, 1.803 ± 0.206; *P* = 7.045×10^−5^) (Fig. 6a).

The population of GABA_A_Rs proteins at the plasma membrane is regulated by a balance of SNARE complex-dependent exocytosis and dynamin-mediated endocytosis^24–26^. To further clarify the mechanism by which protrudin upregulates surface GABA_A_Rs protein levels, the eIPSCs in hippocampal neurons from LV-protrudin and control mice were recorded with exocytosis or endocytosis blocked. eIPSC amplitude did not differ between the LV-protrudin group and LV-GFP group with application of the SNARE-dependent exocytosis blocker TeTx (*n* = 6 neurons from three mice in each group) (Fig. 6b, c and Fig. S2C). However, in the presence of the dynamin-dependent endocytosis blocker dynasore, eIPSC amplitude was high in protrudin overexpression hippocampal slices than in controls (*n* = 6 neurons from three mice in each group) (Fig. 6d, e and Fig. S2D). These results suggest that exocytosis of GABA_A_Rs but not endocytosis might be implicated in the effect of protrudin on membrane distribution.

**Fig. 7:**
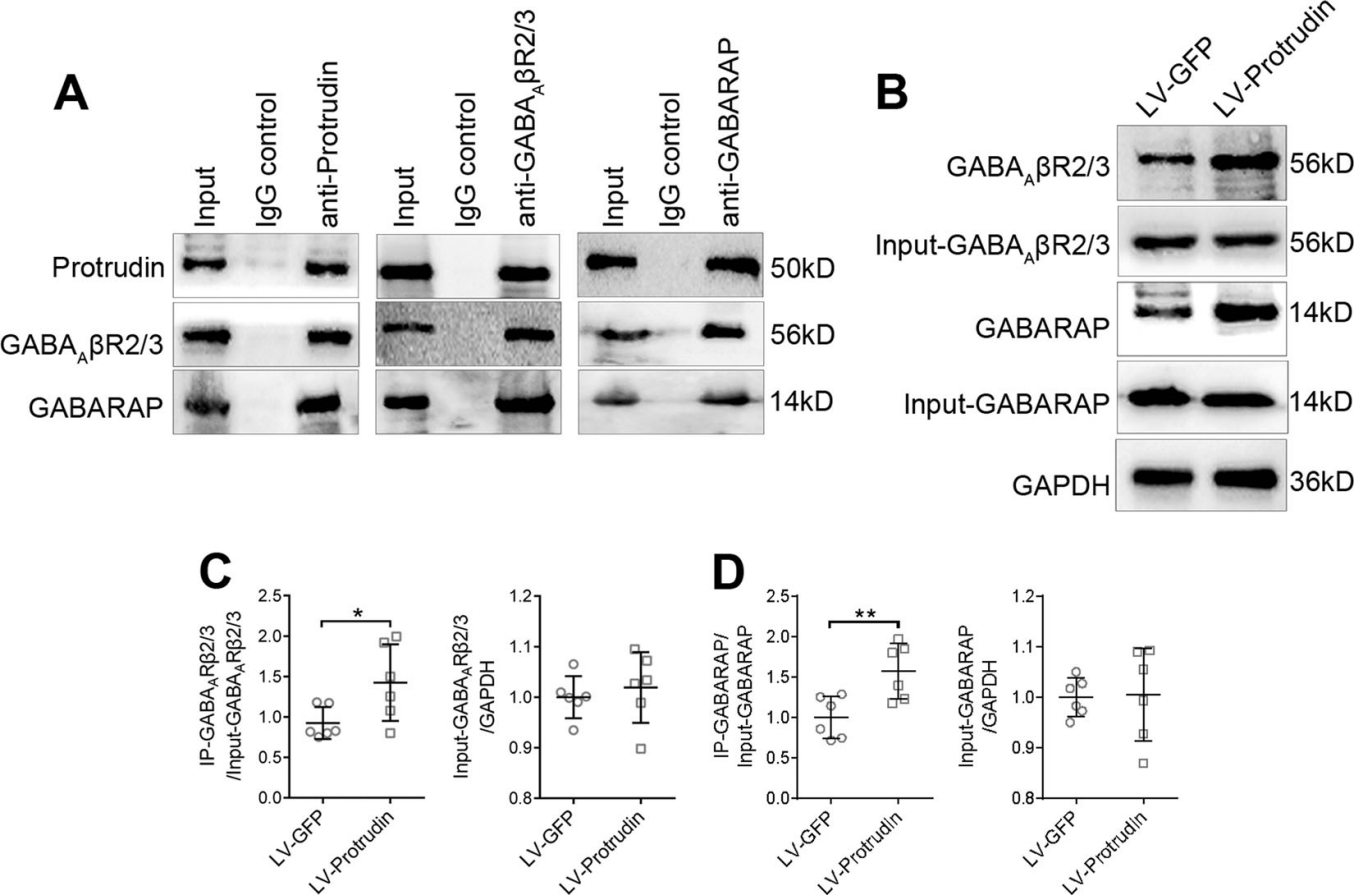
Protrudin interacts with GABA_A_ receptors. **a** Coimmunoprecipitation analysis demonstrated the interactions between protrudin, GABA_A_Rβ2/3, and GABARAP in the hippocampal tissue of epileptic mice. **b** Quantitative coimmunoprecipitation for detecting the binding of protrudin to GABA_A_Rβ2/3 and GABARAP in PTZ-kindled mice. **c** Quantitative analysis showing that the level of coimmunoprecipitated GABA_A_Rβ2/3 was higher in the LV-protrudin group than in the controls, whereas the input of GABA_A_Rβ2/3/GAPDH was not different. **d** Quantitative analysis showing that the level of coimmunoprecipitated GABARAP was higher in the LV-protrudin group than in the controls, and the input of GABARAP /GAPDH was not different. Student’s *t* test, *p < 0.05, **p < 0.01.

GABA receptor-associated protein (GABARAP) is a crucial cytoskeletal protein for the anterograde vesicular delivery of GABA_A_Rs from intracellular stores to the cell membrane^27^. Coimmunoprecipitation analysis revealed protein–protein interactions between protrudin, GABA_A_Rβ2/3 and GABARAP (Fig. 7a) in the hippocampus of PTZ-induced epilepsy mice. Thus, these results indicate that protrudin might affect the transport of GABA_A_Rβ2/3 via an interaction with GABARAP, and the abnormal GABA_A_Rβ2/3 membrane protein could further influence inhibitory postsynaptic transmission in epilepsy.

We further explored whether the interaction of protrudin with GABA_A_Rβ2/3 or GABARAP is altered in a PTZ-kindled epileptic mouse model after protrudin overexpression. In the hippocampus of mice, protrudin overexpression did not alter the initial protein levels of solubilized GABA_A_Rβ2/3 (LV-GFP: 1.0 ± 0.042; LV-Protrudin: 1.019 ± 0.07; *P* = 0.576) or GABARAP (LV-GFP: 1.0 ± 0.038; LV-Protrudin: 1.005 ± 0.092; *P* = 0.912). However, protrudin overexpression increased the level of coimmunoprecipitated GABA_A_Rβ2/3 (LV-GFP: 0.926 ± 0.197; LV-Protrudin: 1.425 ± 0.473; *P* = 0.038) and GABARAP (LV-GFP: 1.0 ± 0.258; LV-Protrudin: 1.569 ± 0.342; *P* = 0.009) (LV-GFP, *n* = 6; LV-Protrudin, *n* = 6) (Fig. 7b–d). These results indicate that protrudin overexpression enhances the interactions between protrudin, GABA_A_Rβ2/3 and GABARAP in epilepsy.

## Discussion

In the present study, we reported that protrudin modulates seizure activity by regulating the GABA_A_Rs-mediated inhibitory synaptic currents and the surface expression of GABA_A_Rs. Low protrudin protein expression was detected in the temporal neocortex of TLE patients and in the hippocampus and temporal cortex of PTZ-kindled and KA-kindled epileptic mouse models. The frequency, duration, and latency of seizures were changed in protrudin overexpressing mice. Furthermore, protrudin overexpression significantly suppressed neural excitability and increased GABA_A_R-mediated inhibitory synaptic transmission, partly due upregulation of the cell surface expression of GABA_A_Rs via an interaction between protrudin, GABA_A_Rβ2/3 and GABARAP. These findings suggest that the low expression levels of protrudin may be implicated in the development of epilepsy.

Protrudin is a protein that contains a FYVE (lipid-binding) domain and a short sequence motif designated FFAT that functions in vesicular transport and neurite formation^4–8^. In our study, we observed that the protrudin protein colocalized with inhibitory synapses in epileptic tissues, indicating that the decreased expression of protrudin may be associated with the development of epilepsy via its role in inhibitory synaptic functions. To further investigate the role of protrudin in epilepsy, behavioral and electroencephalogram abnormalities were identified in lentivirus-mediated overexpression of protrudin in epileptic mouse models. Our results demonstrated that protrudin might exert a protective effect against the development of epilepsy.

Previous reports indicated that protrudin is mutated in the autosomal dominant form of HSP and implied that protrudin has a role in neuronal intracellular membrane trafficking^9^. A common theme of pathogenesis between epilepsies and HSP has been proposed^11^. Some HSP-related proteins are involved in the development of epilepsy^12,28^. Mutations in the gene for KIF5A have recently been reported in HSP^29^, and the KIF5A deletion was identified to cause epileptic seizures^12^. Spastin is also found to be mutated in autosomal dominant pure HSP, and its expression level was decreased in status epilepticus and TLE mouse models^30^. Importantly, KIF5A, spastin, and protrudin are proteins implicated in neuronal vesicular trafficking^5,13^, implicating these proteins in neurotransmitter and/or receptor transport in neurons.

Epileptic seizures are attributed to an imbalance of excitatory and inhibitory synaptic transmission^2^. In the present study, protrudin led to a reduction in neural excitability mainly because of an enhancement in inhibitory synaptic currents, whereas protrudin had no effect on excitatory currents. GABA is a critical inhibitory neurotransmitter in the brain that has an important role in inhibitory neurotransmission via binding to postsynaptic GABA_A_Rs and presynaptic GABA_B_ receptors^23^. GABA_A_Rs strongly control the efficacy of GABAergic inhibition synaptic transmission in the central nervous system. Meanwhile, some famous anti-antiepileptic drugs, benzodiazepines and barbiturates, are GABA_A_ receptor agonists that have been widely used to terminate epileptic seizures and status epilepticus. However, GABA_B_ receptor agonists induce seizure onset^31^. Altering the endogenous expression of protrudin had no effect on IPSC frequency and PPR, indicating that protrudin-mediated inhibitory currents might be regulated by postsynaptic GABA_A_Rs rather than presynaptic GABA_B_ receptors.

GABA_A_Rs are either found at postsynaptic sites where they mediate phasic inhibition or concentrated at extrasynaptic and perisynaptic locations where they mediate tonic inhibition^19^. In our results, protrudin modulated both phasic and tonic inhibitory currents, indicating that protrudin might have an important role in synaptic and extrasynaptic GABA_A_Rs in epilepsy. Pathological conditions in epilepsy have also been shown to remarkably affect the expression of GABA_A_R proteins, which have been implicated in both tonic and phasic inhibition^19,32^.

The most common subunit composition of GABA_A_Rs in the hippocampus is formed by two α1, two β2 or β3, and one γ2 subunit^33^. We observed that upregulation of protrudin increased cell surface GABA_A_Rβ2/3 protein levels without affecting total GABA_A_Rβ2/3 protein expression. Furthermore, our results indicated that the role of protrudin in the upregulation of cell surface GABA_A_Rs protein levels might result from increased GABA_A_Rs exocytosis rather than decreased GABA_A_Rs endocytosis. These findings favor the involvement of protrudin in the intracellular transport of GABA_A_Rs for membrane targeting.

To further investigate the mechanism, the interaction of protrudin with GABARAP and GABA_A_Rβ2/3 in the hippocampus of epileptic mouse models was identified. As the first described GABA_A_R-interacting protein, GABARAP has an essential role in the transport of GABA_A_Rs from intracellular vesicles to the cell membrane, and hence affects the cell surface expression of GABA_A_Rs^34^. Our findings indicated that protrudin-GABARAP-GABA_A_Rβ2/3 complex may play key roles in the forward transport of intracellular GABA_A_Rs to the neural membrane. The integral complex might be damaged in epilepsy, thereby attenuating the surface transport activity of GABA_A_Rs and leading to hyperexcitation of neurons.

In conclusion, we demonstrated that protrudin was decreased in patients with TLE as well as in epileptic mice. Lentivirus-mediated overexpression of protrudin in the mouse hippocampus reduced epileptic susceptibility and activity. Protrudin suppressed neuronal excitability and increased GABAergic synaptic transmission. Furthermore, protrudin upregulated cell surface GABA_A_R protein expression, possibly by affecting GABA_A_R transport through its interaction with GABARAP in epilepsy. We demonstrated for the first time that protrudin plays a crucial role in the development of epilepsy via the regulation of inhibitory synaptic transmission function. This finding may help to identify new target molecules for the treatment of epilepsy.

## Methods

### Human samples

Temporal lobe tissues were randomly obtained from 20 post-surgery patients treated for TLE for whom tissues were available in our brain tissue bank^35–37^. A diagnosis of TLE was determined based on the 2001 International Classification of Epileptic Seizures by the International League Against Epilepsy (ILAE)^38^. The temporal cortical tissues used as control samples were obtained from 12 randomized patients who underwent surgery for trauma-induced intracranial hypertension. The control patients had no history of epilepsy, no exposure to antiepileptic drugs, and no history of neurological or psychiatric disorders. A neuropathological examination confirmed that the temporal neocortex specimens obtained from the control patients were normal. There was no significant difference in age or gender between the TLE and control patients (Table 1).

**Table 1:** Clinical details of TLE patients and control patients.

Cases	Sex	Age (years)	Duration (years)	Seizure type	Preoperative AEDs consumption	Resected tissue	Pathological diagnosis
E1	F	27	14	SGS	PHT, VAP, CZP	RTN	G
E2	F	13	4	SGS	CBZ, TPM, VPA	LTN	NL, ND, G
E3	F	15	3	CPS, SE	CBZ, TPM, LTG	RTN	G
E4	M	47	29	SGS	VPA, PHT, TPM, PB,LTG	RTN	G
E5	M	28	3	SGS	PB, CBZ, VPA	LTN	NL, ND, G
E6	F	38	12	SGS	CBZ, TPM, VPA	LTN	NL, ND, G
E7	F	29	27	SGS	CBZ, VPA, LTG	RTN	NL, G
E8	F	37	7	CPS, GTCS	CBZ, TPM, VPA	LTN	NL, G
E9	M	32	19	CPS	CBZ, TPM, PB,VPA	LTN	ND, G
E10	M	30	10	SGS	CBZ, VPA, PHT	LTN	NL, ND, G
E11	F	11	4	SGS	VPA, CBZ, TPM, LTG	RTN	G
E12	M	22	7	SGS, SPS	VPA, TPM, LTG	LTN	NL, G
E13	F	44	12	CPS	VPA, PHT, LTG	RTN	NL, G
E14	M	33	9	CPS	VPA, PHT, LTG	RTN	G
E15	M	17	3	SGS	PHT, PB, CBZ, LTG	LTN	NL, G
E16	F	14	5	SGS, CPS	VPA, CBZ, PHT, PB	RTN	NL, ND
E17	M	24	3	SGS, CPS	VPA, TPM, OXC	RTN	G
E18	F	33	10	SGS	CBZ, PHT	RTN	G
E19	M	29	14	CPS	VPA, TPM, CBZ, LTG, PHT	LTN	NL, ND, G
E20	M	24	11	CPS	VPA, PHT, CBZ	RTN	NL, G
C1	F	14	0	None	None	LTN	N
C2	F	25	0	None	None	RTN	N
C3	F	32	0	None	None	RTN	N
C4	M	28	0	None	None	RTN	N
C5	M	21	0	None	None	LTN	N
C6	M	54	0	None	None	LTN	N
C7	F	38	0	None	None	RTN	N
C8	M	50	0	None	None	RTN	N
C9	M	43	0	None	None	LTN	N
C10	F	33	0	None	None	LTN	N
C11	M	31	0	None	None	RTN	N
C12	F	15	0	None	None	LTN	N

*AEDs* antiepileptic drugs, *E* epilepsy, *C* control, *F* female, *M* male, *CPS* complex partial seizure, *SPS* simple partial seizure, *SGS* secondarily generalized seizure, *GTCS* generalized tonic clonic seizure, *SE* status epilepticus, *CBZ* carbamazepine, *CZP* clonazepam, *LTG* lamotrigine, *OXC* oxcarbazepine, *PB* phenobarbital, *PHT* phenytoin, *TPM* topiramate, *VPA* valproate, *LTN* left temporal neocortex, *RTN* right temporal neocortex, *NL* neuron loss, *ND* neuron degeneration, *G* gliosis, *N* normal

### Western blot

The hippocampal tissues were isolated from the mice brain after animal behavior experiment. A RIPA protein extraction kit (Beyotime Biotechnology, China) was used for total protein extraction. The Pierce Mem-PER Eukaryotic Membrane Protein Extraction Kit (Pierce, USA) was used to extract membrane protein. Samples boiled with 4× sample buffer at 95°C for 5 min, and then separated on 8–10% sodium dodecyl sulphate-polyacrylamide gel electrophoresis gels and transferred onto polyvinylidene difluoride membranes (Millipore, USA). The target proteins were immunoblotted with primary antibodies overnight at 4°C and then incubated with HRP-conjugated secondary antibodies. The following primary antibodies were used: protrudin (rabbit, Proteintech, USA), GADPH (rabbit, Proteintech, USA), GABA_A_Rβ2/3 (mouse, Millipore, USA), GluR2/3 (rabbit, Millipore, USA) and β-actin (rabbit, Proteintech, USA). The blots were imaged and quantified using a Fusion Imaging System. The quantitative densitometric values of the proteins were normalized to that of GAPDH.

### Immunofluorescence

For animal tissues, brain were post-fixed at 4°C for 24 h. Floating slices (50 mm in thickness) were permeabilized with 0.5% Triton X-100 and blocked in goat serum. The slices were then incubated with primary antibodies, the protrudin (rabbit, Proteintech, USA) and gephyrin (mouse, Santa Cruz Biotechnology, USA) overnight at 4°C followed by incubation with secondary fluorescent conjugated antibodies at room temperature for 2 h. Images were captured using laser-scanning confocal microscopy (Nikon A1 + R Microsystems, Japan) on an Olympus IX 70 inverted microscope (Olympus, Japan) equipped with a Fluoview FVX confocal scanning head. Areas of overlap and fluorescence intensity were analyzed using Image Pro Plus 6.0.

### Intrahippocampal injection of LV

Adult C57/BL6 male mice were obtained from the Experimental Animal Center of Chongqing Medical University. Intrahippocampal injection of LV was performed using a stereotaxic apparatus (Stoelting Co. Ltd Wood Dale, IL, USA). In brief, the mice were anesthetized with pentobarbital (80 mg/kg, intraperitoneal) and placed on stereotaxic apparatus. The reference points were bregma for the anterior-posterior axis, the midline for the medial-lateral axis and the dura mater for the dorsal-ventral axis with a tooth bar. LV particles were injected into the dorsal hippocampus through a glass pipette (0.2 μl/min) attached to a glass microsyringe^36^. The mice were allowed to recover for 2 weeks after the vector injections.

### Animal behaviors in epilepsy

Two weeks after the intrahippocampal injection of LV, the mice were kindled by PTZ or KA to measure epilepsy susceptibility. This PTZ-induced model was performed according to the methods described in a previously study^39^. C57/BL6 mice were administered an intraperitoneal injection of PTZ (Sigma-Aldrich, USA) (35 mg/kg) every other day and then observed for 60 min after each injection. Behavioral seizure scores were assessed based on the Racine standard scale, as follows: Grade 0 indicated arrest, wet dog shaking, and normal behavior; Grade 1 indicated facial twitches (nose, lips, and eyes); Grade 2 indicated chewing and head nodding; Grade 3 indicated forelimb clonus; Grade 4 indicated rearing and falling on forelimbs; and Grade 5 indicated imbalance and falling on the side or back^40^. Only fully induced seizures (Grades 4 and 5) were evaluated to determine the number of seizures and the latency period. The controls was not performed at least three consecutive seizures with a score of 4 or 5.

Mice were injected with KA as previously described^41^. In brief, 2 weeks after the intrahippocampal injection of LV, the mice were anesthetized and slowly injected with KA (Sigma-Aldrich, USA) (1 nmol in 50 nl) into dorsal hippocampus via a guide cannula under stereotaxic guidance. At 1 h after the onset of KA-induced status epilepticus (SE), diazepam (10 mg/kg, i.p.) was administered to suppress SE. After SE successful induction, the chronic epileptic animal model was confirmed for 30 days. During this chronic phase, mice exhibited at least one spontaneous recurrent seizure with motor manifestations was included in the epilepsy group, and mice that did not show spontaneous recurrent seizures were used as controls. Multichannel electroencephalogram (EEG) recording was performed to quantify the EEG seizures using the OmniPlex D Neuronal Data Acquisition System (Plexon, Dallas, TX, USA) as previously described^35,36^. The signals were preamplified (1000×), filtered (0.1–1000 Hz), and digitized at 4 kHz. EEG SLEs were characterized by high frequencies (> 5 Hz), high amplitudes (> 2 times the baseline), and durations of > 5 s^42^.

### Patch clamp recordings

Brain slices were prepared as previously reported^43,44^. In brief, C57/BL6 mice were anesthetized with pentobarbital. Brain slices (300 μm) were prepared with a Leica (Germany) VP1200S Vibratome and incubated in ACSF (119 mm NaCl, 26 mm NaHCO_3_, 2.5 mm KCl, 1.3 mm MgCl_2_, 1.25 mm NaH_2_PO_4_, 2 mm CaCl_2_ and 25 mm glucose, pH 7.4, 310 mOsm) bubbled with 5% CO_2_ and 95% O_2_ for at least 1 h at room temperature before recording. Whole-cell recording was performed as described previously^45^. Glass microelectrodes (Sutter, USA) were shaped by a pipette puller (P-97, Sutter, USA) to a final resistance of 3–5 MΩ when the pipette was filled with internal solution. A multi-clamp 700B amplifier (Axon, USA) was used for the recordings. Signals were sampled at 10 kHz and filtered at 2 kHz. A stable baseline was obtained for at least 5 min prior to recording and data were discarded when the access resistance (15–20 MΩ) was changed by 20% at the end of recording. Mini Analysis 6.0.1 (Synaptosoft) and Clamp Fit 10.3 software (Axon, USA) were used to analyze the recorded data.

Neuronal electrophysiological changes were detected in hippocampus slices of PTZ- and KA-kindled mouse models and of Mg^2+^-free-ACSF epilepsy cell model. For AP recording, glass pipettes were filled with the following internal solution: 17.5 mm KCl, 0.5 mm EGTA, 122.5 mm k-gluconate, 10 mm HEPES, and 4 mm ATP, pH adjusted to 7.2 with KOH. The internal solution used to record EPSCs contained: 17.5 mm CsCl, 10 mm HEPES, 4 mm ATP, 0.5 mm EGTA, 132.5 mm Cs-gluconate, and 5 mm QX-314. The internal solution used to record IPSCs contained: 100 mm CsCl, 1 mm MgCl_2_, 1 mm EGTA, 30 mm *N*-methyl-d-glucamine, 10 mm HEPES, 5 mm MgATP 0.5 mm Na_2_GTP and 12 mm phosphocreatine. Tetrodotoxin (TTX, 1 μm), bicuculline (10 μm) were added to ACSF to record mEPSCs when the membrane was voltage-clamped at − 70 mV. TTX(1 μm), DNQX(20 μm), and APV(40 μm) were added to ACSF to record mIPSCs at −70 mV^36^. The method of sIPSCs recording was as same as mIPSCs without adding TTX in ACSF. sIPSCs were recorded in the present of gabazine (SR95531) (0.5 μm) or picrotoxin (10 μm) in ACSF^20^. eIPSCs were collected in the present of DNQX (20 μm) and APV (40 μm) at a holding potential of − 70 mV. eIPSCs were generated with a 40 μs pulse (0.1 Hz) from a stimulated isolation unit controlled by an AMPI generator (Master-8, USA). A bipolar stimulation electrode (FHC) was located ~ 100 μm rostral to the recording electrode in the same region^46,47^. eIPSCs were collected in the present of DNQX (20 μm) and APV (40 μK) at a holding potential of −70 mV. PPR recordings were obtained using a paired-pulse protocol of two stimuli at an inter-pulse interval of 50 ms. PPR values were defined as the ratio of the second peak amplitude to the first peak amplitude. To clarify the mechanism by which protrudin affects GABA_A_Rs membrane expression, the exocytosis blocker TeTx (0.1 m) and the endocytosis blocker dynasore (80 mm) were added to the internal solution for eIPSCs recordings^48^.

### Coimmunoprecipitation assays

Coimmunoprecipitation and quantitative coimmunoprecipitation assays were described previously^48^. The extracts obtained from hippocampal tissues from the PTZ-induced epileptic mice were prepared with anti-protrudin(rabbit, Proteintech, USA), anti-GABA_A_Rβ2/3 (rabbit, Millipore, USA), anti-GABARAP (rabbit, Proteintech, USA) or IgG (rabbit, Abcam, USA) (Control) at 4°C for 12 h before they were incubated with protein A/G agarose beads (Beyotime, China) at 4°C overnight. The immunoprecipitated mixture was immunoblotted with above antibodies.

### Study approval

All protocols of animal studies were approved by the Commission of Chongqing Medical University for the ethics of experiments on animals and conducted in accordance with international standards. All procedures of human samples collections were formally consented to by the patients or their lineal relatives and approved by the Ethics Committee of Chongqing Medical University according to the tenets of the Declaration of Helsinki.

### Statistical analysis

All averaged data are presented as the means ± SD, and all graphs were prepared using GraphPad Prism 4 software (La Jolla, CA). Exact mean, SD, and *P* values of electrophysiological results have been stated in Table S1. For independent-samples, Student’s *t* test was used. Repeated measures ANOVA followed by post hoc *t* tests was used to measure differences between two groups at multiple time points. The *χ*^2^ test was used to compare gender differences between the patients in the TLE and control groups. *p* < 0.05 and 0.01 were considered to indicate statistical significance and are indicated as *p < 0.05 or **p < 0.01, respectively. All tests were two-sided. *n* indicates the number of cells/slices or independent experiments and was used to calculate the degree of freedom. All samples included in each experiment were analyzed in triplicate.

## Supplementary information

Supplementary Figure Legends

Figure S1

Figure S2

Supplementary Information
