# Supplementary material for: Protrudin modulates seizure activity through GABAA receptor regulation
Source: Cell Death Dis. 2019 Nov 27;10(12):897. doi: 10.1038/s41419-019-2118-8 (PMC6879747; doi:10.1038/s41419-019-2118-8)
Supplement: Supplementary file 1 — Supplementary Figure Legends [file 41419_2019_2118_MOESM1_ESM.docx]

**Fig. S1 Effects of protrudin on electrophysiology in the hippocampus of Mg2+-free-ACSF epilepsy cell model.**

A, The frequency of spontaneous AP was compared between the LV-protrudin group and the control group. Bar, 12.5mV, 1.25s. B, Protrudin had no significant effect on the amplitude or frequency of mEPSCs. C, D, Quantitative analysis of mIPSC (C) and sIPSC (D) amplitude and frequency in the hippocampus between the LV-protrudin group and the control group. E, F, Quantitative analysis of the phasic (E) and tonic (F) current amplitude between the LV-protrudin group and the control group. Bar (B-F), 10pA, 1.25s. Student’s t test, **p<0.01.

**Fig. S2 Effects of protrudin on IPSCs in the hippocampus of Mg2+-free-ACSF epilepsy cell model.**

A, eIPSCs from hippocampal pyramidal cells in protrudin overexpressing mice. B, Overexpressing protrudin did not alter the PPR values. C-D, Representative traces of eIPSCs in the LV-protrudin group and control group after treatment with TeTx (C) or dynasore (D). Bar, 20pA, 12.5s. Student’s t test, **p<0.01.
