# Supplementary figures and images for: Protrudin modulates seizure activity through GABAA receptor regulation
Source: Cell Death Dis. 2019 Nov 27;10(12):897. doi: 10.1038/s41419-019-2118-8 (PMC6879747; doi:10.1038/s41419-019-2118-8)

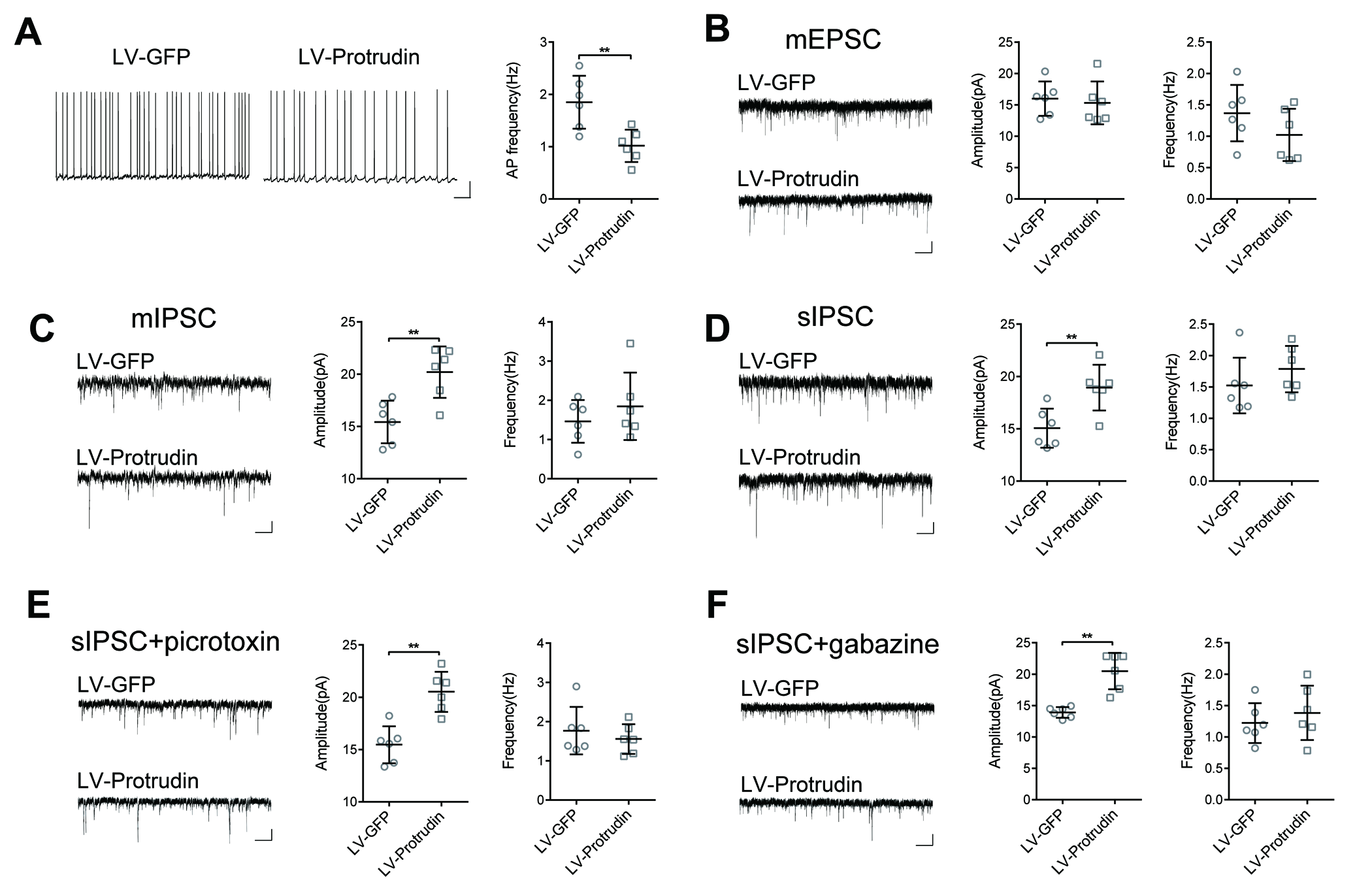

Supplement: Supplementary file 2 — Figure S1 [file 41419_2019_2118_MOESM2_ESM.tif]

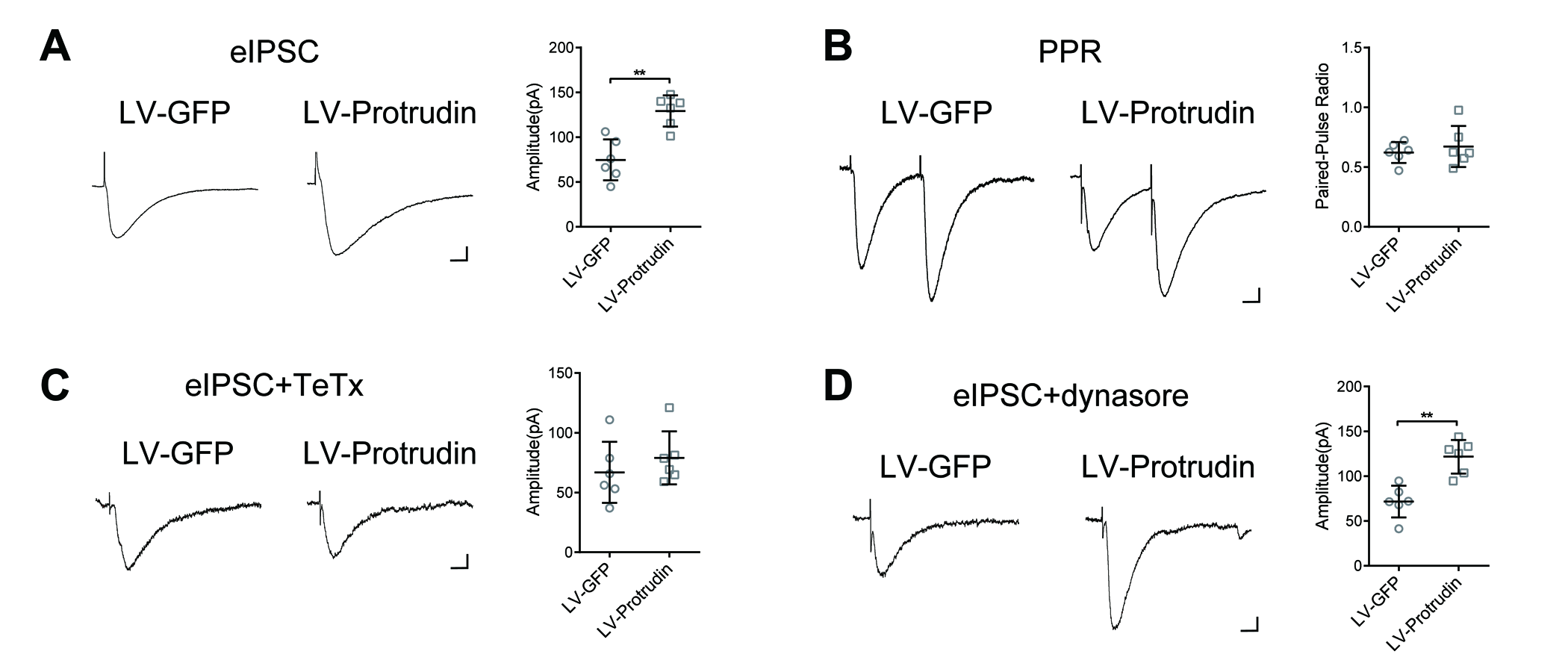

Supplement: Supplementary file 3 — Figure S2 [file 41419_2019_2118_MOESM3_ESM.tif]
