# Supplementary material for: Protrudin modulates seizure activity through GABAA receptor regulation
Source: Cell Death Dis. 2019 Nov 27;10(12):897. doi: 10.1038/s41419-019-2118-8 (PMC6879747; doi:10.1038/s41419-019-2118-8)
Supplement: Supplementary file 4 — Supplementary Information [file 41419_2019_2118_MOESM4_ESM.docx]

**Table S1. Mean, SD and *P* values of electrophysiological results.**

|  | PTZ-kindled model | | | KA-kindled model | | | Mg^2+^-free model | | |
| --- | --- | --- | --- | --- | --- | --- | --- | --- | --- |
|  | LV-GFP | LV-Protrudin | *P* | LV-GFP | LV-Protrudin | *P* | LV-GFP | LV-Protrudin | *P* |
| AP | 1.773±0.562 | 0.925±0.251 | 0.007 | 1.739±0.421 | 1.021±0.279 | 0.006 | 1.849±0.507 | 1.017±0.308 | 0.006 |
| mEPSC amplitudes | 16.073±4.675 | 16.436±3.547 | 0.883 | 14.512±2.227 | 14.308±1.894 | 0.868 | 15.993±2.735 | 15.305±3.412 | 0.708 |
| mEPSC frequency | 1.126±0.595 | 1.216±0.401 | 0.766 | 1.098±0.520 | 1.316±0.321 | 0.404 | 1.367±0.449 | 1.021±0.416 | 0.197 |
| mIPSC amplitudes | 14.893±1.189 | 19.815±2.480 | 0.001 | 16.074±0.847 | 20.311±2. 747 | 0.004 | 15.432±2.046 | 20.200±2.461 | 0.004 |
| mIPSC frequency | 1.233±0.433 | 1.769±0.601 | 0.107 | 1.635±0.142 | 1.783±0.261 | 0. 249 | 1.464±0.545 | 1.848±0.861 | 0.378 |
| sIPSC amplitudes | 13.800±1.819 | 18.800±2.606 | 0.003 | 14.753±1.692 | 17.748±2.505 | 0.035 | 15.060±1.865 | 18.933±2.184 | 0.008 |
| sIPSC frequency | 1. 447±0. 317 | 1.359±0. 526 | 0. 733 | 1.445±0.248 | 1.569±0.294 | 0. 452 | 1.522±0.445 | 1.784±0. 369 | 0. 293 |
| Tonic amplitudes | 15.092±1.311, | 21.209±1.618 | 2.941×10^-5^ | 17.283±2.201 | 20.920±2.212 | 0.017 | 13.903±0.860 | 20.500±2.907 | 3.325×10^-4^ |
| Tonic frequency | 1.507±0.433 | 1.306±0.442 | 0. 443 | 1.406±0.403 | 1. 512±0. 423 | 0. 667 | 1.222±0.317 | 1.384±0.434 | 0. 477 |
| Phasic amplitudes | 14.258±1.497 | 19.731±3.333 | 0.004 | 15.132±1.333 | 19.313±1.513 | 4.778×10^-4^ | 15.459±1.763 | 20.517±1.916 | 7.710×10^-4^ |
| Phasic frequency | 1.777±0. 237 | 1.617±0.420 | 0. 435 | 1.365±0. 376 | 1.664±0.611 | 0. 331 | 1.769±0.603 | 1.556±0.378 | 0. 483 |
| eIPSC | 72.333±14.446 | 120.25±14.427 | 1.855×10^-4^ | 72.417±18.049 | 134.05±17.178 | 1.211×10^-4^ | 74.717±22.783 | 129.167±17.419 | 9.072×10^-4^ |
| PPR | 0.696±0.190 | 0.633±0.098 | 0.483 | 0.551±0.147 | 0.572±0.127 | 0.795 | 0.623±0.087 | 0.673±0.171 | 0.541 |
| eIPSC-TeTx | 73.45±26.917 | 79.983±26.804 | 0. 682 | 60.783±16.87 | 84.017±27.928 | 0.112 | 66.917±25.559 | 79.033±22.185 | 0. 401 |
| eIPSC- dynasore | 71.55±18.701 | 128.6±20.828 | 5.434×10^-4^ | 84.583±16.015 | 127.367±20.261 | 0.002 | 71.683±17.709 | 121.75±18.763 | 7.763×10^-4^ |

AP, action potential; EPSC, excitatory post-synaptic currents; IPSC, inhibitory post-synaptic currents; mEPSC, miniature EPSC; sIPSC, spontaneous IPSC; eIPSC, evoked IPSC; PPR, paired-pulse ratio; PTZ, pentylenetetrazol; KA, kainic acid.
